# Grand Challenge Animal Reproduction-Theriogenology: From the Bench to Application to Animal Production and Reproductive Medicine

**DOI:** 10.3389/fvets.2017.00114

**Published:** 2017-07-17

**Authors:** Ahmed Tibary

**Affiliations:** ^1^Department of Veterinary Clinical Sciences, Center for Reproductive Biology, Washington State University, Pullman, WA, United States

**Keywords:** theriogenology, reproductive medicine, reproductive biology, artificial breeding

## Introduction

Reproductive physiology and procreation has always fascinated human kind. Therefore, it is not surprising that scientific research in reproduction is one of the oldest and most established field in biology. Advancement in reproductive sciences has been possible because of the curiosity of scientists of various backgrounds (biologists, animal scientists, and veterinarians). At the turn of the twentieth century, advances in reproductive research were mostly driven by needs for improved animal production and prevention of venereal diseases. The body of knowledge in animal reproduction has seen an exponential growth in the last 50 years. In recent years, the field of study expanded beyond laboratory species and production animals to include wildlife conservation and management.

As this field of research grew, scientists felt the importance of organizing in international societies dedicated to this area. One of the oldest of these societies is the Society for the Study of Reproduction. In the veterinary field, reproductive physiology and pathology became known as “Theriogenology” thanks to the efforts of the founding members of a veterinary specialty under the name of the American College of Theriogenologists, recognized in 1971 by the American Veterinary Medical Association as an integral part of the veterinary curriculum (1). Similar specialty colleges were also started in Europe (European College of Animal Reproduction), Australia, and New Zealand (College of Veterinary Scientists, Animal Reproduction). In addition to these specialty colleges, other international societies have emerged including Society for Theriogenology, International Embryo Transfer Society, European Society for Domestic Animal Reproduction, and European Society for Small Animal Reproduction. All these society have now well-established regular meetings to provide a forum for communication of recent research and their application to the health and welfare of animals. This paper attempts to highlight some of the major milestones and challenges in reproductive research.

## Reproductive Physiology and Pathology in the Male

Tremendous progress has been achieved in understanding of sexual differentiation, testicular differentiation, and function (2–4). Early endocrine studies shed light on early testicular development and puberty. Clinical studies in the domestic animal led to the development of the field of applied clinical andrology (5). Studies incorporating clinical methods for prediction of fertility proved to be useful in the screening of males for optimal fertility in production animals and helped identify potential problems in individual animals with high genetic value. Breeding soundness examinations are now standardized techniques used in veterinary practice in almost all domestic animals. However, the prediction of fertility on a case-by-case basis remains challenging despite the development of a large number of clinical, histological, and molecular techniques (6, 7).

One the most important areas of research in male reproduction is the understanding of factors affecting spermatogenesis and sperm production. Traditionally, this has been approached through experimental designs involving live animals. The development of techniques such as specific molecular probes and xenografting of testicular tissues opened a new era of study on factors affecting spermatogenesis (8–11).

## Male Gamete Preservation and Artificial Insemination

Artificial insemination is recognized as the reproductive technology that had the most impact on animal reproduction. Although semen collection and preservation has long been established in several species, challenges still exist in others (camelids, wildlife). Sperm preservation in liquid (chilled) or frozen (cryopreservation) form continues to be a challenge because of tremendous individual male variation particularly in some species (i.e., equine, camelids). Molecular techniques have allowed scientists to detect changes during preservation which affect fertility (5). Recently, a focus was put on reactive oxygen species and how they alter sperm function (12).

Advances in sperm technologies include the introduction of sperm sexing for gender selection and the preservation of epididymal sperm. The limitation of use of sex-sorted semen imposed reconsideration of established artificial insemination parameters such as required number of sperm per insemination (13). Preservation of epididymal sperm is one of the most important aspects in preservation of genetic material from valuable terminally ill or deceased animals (wild and domestic species) (14–18). This new approach to breeding raises fundamental physiological questions on the role of seminal plasma in fertility (19–21).

Other significant advances in sperm technologies include cryopreservation by vitrification (22, 23), refreezing of spermatozoa (24–27), and production of spermatozoa from frozen testicular tissue (28, 29). Development in spermatogonia stem cell (SSC) culture opens a new era in the understanding of testis function and male fertility (2, 30–32). Transfer of SSC will eventually revolutionize our breeding strategies and preservation of genetics (33).

## Reproductive Physiology in the Female

Without doubt one the most important achievements in the study of reproductive physiology in the female is the development of protocols for synchronization of estrus and ovulation. These protocols proved to be of great values in production animals. Fixed-time artificial insemination is now a standard procedure for dairy and beef production as well as for large-scale sheep and goat operations (34–36). Estrous and ovulation synchronization protocols are also an integral part of embryo transfer programs. These techniques have become possible through advances in our understanding of the endocrinology of the female reproductive cycle and also through the introduction of *in vivo* monitoring of follicular dynamics through the use of ultrasonography (37). The characterization of follicular wave dynamics used initially in the bovine has become a standard approach to any study on the reproductive pattern in the female and has been adopted to study other species (i.e., camelids) (38). As ultrasonography technology advances, its uses in reproductive biology multiply. The development of Doppler ultrasonography provided a better characterization of ovulation, corpus luteum development, and blood flow to the uterus (39, 40).

Another area of advancement in female reproductive biology is the study of maternal recognition of pregnancy (MRP) (41, 42). Major advances have been realized in ruminants, shedding light on mechanisms involved in embryo elongation and trophoblast/endometrium cross talk during MRP (43, 44). Molecular and genomics techniques provided insights on this mechanism and its implication in infertility and early embryonic loss (45). However, challenges still exist in deciphering MRP mechanisms in several species including equine (46, 47) and camelids (38).

## Female Gamete Preservation

Oocyte preservation has been lagging behind comparatively to sperm. However, in recent years and with the development of ultrasound-guided follicular aspiration, oocytes are now routinely and repeatedly collected from valuable females and either stored or fertilized. Fertilization *in vivo* (oocyte transfer) or *in vitro* through techniques such as intracytoplasmic sperm injection or *in vitro* fertilization (IVF) is now commonplace in ruminants (48). Development of these technologies has seen a surge in the equine recently (49, 50). Oocyte cryopreservation has been challenging; however, newer techniques of verification show great promise (51). *In vivo* and *in vitro* production of oocytes from preserved/transplanted ovarian tissue is a novel technique used for the preservation of reproductive ability and could be an asset for the preservation of endangered species or genetic material from animals (52, 53). The effect of all these technologies on oocyte activation and capacity to develop into a viable embryo after fertilization is a subject of study using advanced molecular and genomic techniques (54).

## Embryo Technology

*In vivo* and *in vitro* production has become commonplace for several species. In ruminants, and more recently in camelids, advancement in protocols for ovarian superstimulation allowed reduction of cost and increased use of these technologies in production systems. In cattle, embryo transfer activity is progressively moving toward the use of *in vitro* produced embryo *via* IVF or somatic cell nuclear transfer (SCNT). Pregnancies obtained from *in vitro* produced embryos and particularly those by SCNT have been challenging due to increased pregnancy loss and increased rate of abnormalities of the placenta (hydrops) or fetus (abnormal large offspring syndrome). SCNT has been reported in almost all domestic animal species. However, adoption of this technique for large-scale production remains challenging due to the great variability of results due to increased early pregnancy loss and abnormal pregnancies. Genomic studies showed a great difference in gene expression between embryos derived *in vivo* and those produced *in vitro* which could explain these abnormalities (55–57).

Embryo cryopreservation has become common place in the majority of ruminant production systems but still faces challenges in other species (equine, camelid). New techniques such as vitrification and dehydration prior to freezing are being developed and show some promise particularly in the equine and camelid species (58, 59).

Embryo manipulation allowed development of power tools such as testing for genetic disorders on embryo biopsies prior to transfer. Genetically engineered animals have been the goal of several studies for various reasons. This technology progressively moved from relatively crude techniques of production of transgenic animals (60, 61) to more sophisticated genome editing techniques such as clustered regularly interspaced short palindromic repeats (62, 63). These techniques will be great tools for the production of animals with specific genes of interest.

## Reproductive Medicine

Reproductive medicine research has focused on two paradoxical goals: contraception and enhanced fertility.

Effective non-surgical contraception has been a goal for many domestic and feral or wild species. Several approaches have been considered including immunization against zona pellucida and GnRH. However, these techniques proved to be ineffective in some species and not practical for large-scale use particularly in wild or feral animals (64–66). Another challenge in population control in the wild is the need for an effective method of contraception without alteration of normal reproductive behavior that often regulates the herd social structure. A challenge grant has been offered to find a safe and effective (permanent), single-dose non-surgical sterilant for both genders of cats and dogs (http://www.michelsonprizeandgrants.org/). This has generated new interest and development of new approaches to contraception.

Enhanced fertility continues to be one of the most important aspects of production animal clinical services. The cattle dairy industry is the most concerned, as there is a trend toward poor reproductive efficiency with increased production (67). The first challenge in this industry was the inability to adequately detect estrus and reinseminate cows in a timely manner. This was in part due to human error in management and also due to changes in the reproductive biology of the high-producing cow. The interaction between metabolic activity and circulating hormone patterns is responsible for poor expression of estrus, increased rate of anovulatory cycles, and increased early embryonic loss. Strategies to improve the hormonal profiles are possible, but they are always limited by regulations on the use of hormones in food-producing animals. The interaction between metabolic disorders, reproductive function, and susceptibility to uterine infection has been studied extensively in dairy cattle (68–72).

Other factors involved in reduced fertility in production animals that remains a challenge to the scientist and practitioner include environment and interaction with systemic disease. Heat stress has long been identified as a major limiting factor in reproduction. Molecular and genomics studies have shown a profound effect of heat stress on oocyte and embryo quality (68, 73). These studies represent a model of study as climatic changes experienced by the planet are bound to continue to have an effect on reproduction of both wild and domestic species.

Reproductive medicine/Theriogenology is also an individual animal practice. Several clinical problems have been studied to provide the best care for subfertile animals or animals with high-risk pregnancies. In addition to the common use of imaging in reproduction, new surgical and non-surgical techniques are being developed to diagnose and treat causes of infertility. The main species that have benefited from such techniques are small animals, equine, and camelid. The combination of established techniques such as endometrial biopsy, culture, and cytology with molecular techniques has allowed substantial advances in our understanding of the pathophysiology of endometritis and led to the development of methods for diagnosis, prevention, and treatment (74, 75).

The effect of age on follicular dynamics and endometrial degenerative changes has been studied primarily in mares (76–78). Stem cell therapy for these degenerative changes is being investigated (79, 80).

Advances in our understanding of the endocrinology of pregnancy and ultrasonographic evaluation of the fetus and placenta allowed a more efficient way for the evaluation of high-risk pregnancy in mares. An experimental model for ascending placentitis in this species allowed scientists to establish protocols for the diagnosis, monitoring, and treatment of this common cause of abortion and premature delivery (81–84).

Another area of critical importance in reproductive medicine is the diagnosis and prevention of abortion. Several infectious causes of abortion in ruminants are zoonotic and present serious health risk for humans (85). Strategies for rapid diagnosis of infectious causes of abortion have become available with the introduction of highly specific and sensitive molecular techniques (86). Studies on the pathophysiology of some of these diseases allowed a better understanding of the host–pathogen interaction and the development of congenital abnormalities. This has been useful recently in the discovery and study of a new disease “Schmallenberg” (87).

Reproductive toxicology has generated tremendous interest from various researchers. In addition to the traditional toxins (i.e., mycotoxin, plants) known to cause reproductive disorders (infertility or abortion), steroid disruptors have been a great concern for both animal and human health (88, 89).

## Conclusion

The primary goal of this article was to highlight the complexity and variety of areas of research in reproductive physiology and medicine. As methods for the study of reproduction have increased in sophistication, a huge amount of information has been generated. This creates a challenge for the scientific community and the practitioner as it becomes very difficult to translate some of the discoveries into application to medicine. This concern has already been stated almost a decade ago by Sirard who wrote “…too many publications are now reporting observations with little understanding of their value or how they fit in the big picture…” (90). It is important that scientists keep this in mind when developing training programs for future reproductive physiologists and theriogenologists. Multidisciplinary collaborations between bench scientists and clinical researchers will be more and more important. One aim of the Frontiers in Veterinary Science specialty section on Animal Reproduction/Theriogenology will be to bridge the gap between fundamental science and application to animal health and reproduction.

## Author Contributions

The author confirms being the sole contributor of this work and approved it for publication.

## Conflict of Interest Statement

The author declares that the research was conducted in the absence of any commercial or financial relationships that could be construed as a potential conflict of interest.
